# Ambroxol hydrochloride spray (Luo Runchang®) in the treatment of acute respiratory infectious diseases: a prospective, multicenter, open label, randomized controlled study

**DOI:** 10.3389/fped.2024.1380189

**Published:** 2024-09-05

**Authors:** Lu Cheng, Min Liu, Rong Wang, Sufen Cao, Rui Li, Bo Su, Hongyan Wei, Haijuan Yang, Lingyun Hou, Chunyu Geng, Yuling Han, Tianrui Yang

**Affiliations:** ^1^ Department of Pediatrics, Children's Hospital Affiliated to Shandong University, Jinan Children's Hospital, Jinan, Shandong, China; ^2^ Department of Pediatrics, Jinan City People’s Hospital, Jinan, Shandong, China; ^3^ Department of Pediatrics, Cangzhou People’s Hospital, Cangzhou, Hebei, China; ^4^ Department of Pediatrics, The People's Hospital of Langfang City, Langfang, Hebei, China; ^5^ Department of Pediatrics, Linyi People’s Hospital, Linyi, Shandong, China; ^6^ Department of Pharmacy, Houston Methodist Hospital, Houston, TX, United States

**Keywords:** ambroxol, lower respiratory tract infection (LRTI), ambroxol hydrochloride, pediatric medicine, respiratory infection

## Abstract

**Purpose:**

Cough and sputum are the most common clinical symptoms of acute respiratory tract infection. Ambroxol is a mucolytic expectorant commonly used in clinical practice. This study aimed to evaluate the efficacy, safety, and compliance of ambroxol hydrochloride spray (Luo Runchang ®) for the treatment of acute respiratory tract diseases in children.

**Methods:**

This was a multicenter, open-labeled, randomized controlled study. The experimental group received ambroxol hydrochloride oral sprays, and the control group received ambroxol hydrochloride oral solutions. The primary endpoint was the change in cough symptom scores from baseline. Secondary endpoints include changes in cough severity score, quality of life, adherence, and adverse events.

**Results:**

A total of 154 subjects were randomized and included in the analysis. The mean change of total cough symptom score of the spray group at the end of treatment was −4.7 (1.54) compared to −4.2 (1.62) in the solution group (*P* = 0.0005). The mean change of cough severity score was −5.7 (2.09) in the spray group compared to −5.2(2.04) in the solution group (*P* = 0.012). Quality of life scores significantly improved in the spray group (*P* < 0.0001) compared to the oral solution group. Medication adherence markers were significantly better in the spray group (*P* < 0.0001). The incidence of adverse events in the experimental group (1.33%) was lower than that in the control group (6.33%), but the difference between the groups was not statistically significant.

**Conclusion:**

Ambroxol hydrochloride spray significantly improved cough symptom score, cough severity score, and quality of life score compared to ambroxol hydrochloride oral solution.

## Introduction

Lower respiratory infections are a global and persistent public health problem. In the Global Burden of Disease study published by the World Health Organization in 2019, lower respiratory infections were listed as one of the leading cause of infectious diseases, causing approximately 105 million disabilities, a greater burden of disease than human immunodeficiency virus infections, malaria, cancer, or diabetes (1, 2). Acute respiratory tract infection is the most common infectious disease in children. Acute lower respiratory tract infection is the first cause of death in children under 5 years old. It seriously threatens the life and health of children and causes a serious burden on families and society (3). Cough and sputum are the most common clinical manifestations in children with respiratory infections. The essence of cough is the protective reflex of the respiratory tract against various irritations. It is also one of the most common symptoms of respiratory system disease and the reason for seeking medical treatment (4). A previous study has shown that more than 75 percent of children had more than five physician visits per year due to chronic cough, and 14 percent had more than 15 visits (5).

The main pathological mechanism of sputum is hypersecretion of airway mucus and obstruction of mucus clearance (6). During an acute infection, airway epithelium is damaged, inflammatory cells are released, and a large number of secretory cytokines are produced by the body to act on secretory cells, which leads to the proliferation of airway glands and goblet cells, and increase mucus production and secretion. The hypersecretion of mucus leads to the retention of airway mucus and the symptoms of cough and sputum (7, 8).

Airway mucus-cilia clearance is one of the critical defense mechanisms of the respiratory system (9). The main function of ciliary epithelial cells is to clear mucus. Ciliary oscillations clear mucus in a cyclic process. Through the coordinated oscillations of cilia, mucus in the airway is pushed into the oropharynx, coughed up, or swallowed. Respiratory tract infection can cause ciliary dysmotility, resulting in reduced sputum discharge and increased sputum volume in the respiratory tract (10). Expectorant drugs can improve the efficiency of cough in clearing airway secretions. The mechanisms of action for expectorant medications include: increasing secretion output, reducing secretion viscosity, and enhancing ciliary clearing function. Ambroxol, a metabolite of bromhexine *in vivo*, is a commonly used mucolytic expectorant. It not only can depolymerize acidic polysaccharides to reduce mucous viscosity but can also change the rheological properties of secretions by acting on ciliated epithelial cells, improve the mucous elimination in ciliated and non-ciliated areas of the respiratory tract, reduce the adhesion of sputum and ciliate, and increase the mucous ciliate removal rate. Through these mechanisms, ambroxol helps to promote sputum discharge (11–13).

Ambroxol has been widely used in treating acute and chronic respiratory diseases since the 1970s and has a good safety profile in adults and children. Ambroxol has a variety of dosage forms, among which injection, oral solution, syrup, aerosol inhalant, etc., are clinically used for children's expectorant treatment. A large number of clinical reports have shown that ambroxol combined with anti-infective drugs is effective and safe in the treatment of children with respiratory tract infections (14).

Expectorant drugs still have the following problems. In China, only a few dosage forms have approved indications for pediatric patients, and the administration of medication to children is challenging, leading to poor adherence and an increased risk of inaccurate dosage. In order to facilitate the use of medicines for children, Shandong Yuxin Pharmaceutical Co., LTD., a wholly owned subsidiary of Shandong Luoxin Pharmaceutical Group Stock Co., LTD., has developed China's first children's spray expectorant. The registered class is 2.4 new drugs, suitable for 2–6 years old children with thick sputum and difficult expectoration, easy to use, and accurate dosage. It is expected to solve the problem that the existing dosage forms cannot meet the clinical needs.

## Methods

### Study design

This was a multi-centered, prospective, open-label, randomized-controlled study conducted at 5 centers in China from November 2021 to August 2022. Patients were randomized using the Interactive Web Response System after written informed consents were obtained from the parents. Patients were stratified based on the severity of the disease. Patients were randomized 1:1 to receive the ambroxol spray or the ambroxol solution. The treatment group received spray 2 inhalations (15 mg) three times a day and the control group received the ambroxol solution 2.5 ml (7.5 mg) three times a day if the patient was aged 2–5-year-old or 5 ml (15 mg) three times a day if the patient was 6-year-old. Patients’ baseline characteristics are obtained upon initiation of therapy. Patients are followed for 3 to 7 days post-initiation depending on time of discharge.

The study protocol was approved by independent ethics committees or institutional review boards at every treatment center and was conducted in accordance with the ethical principles of the Declaration of Helsinki.

### Participants

Patients are included if they are (1) aged 2–6 years old, (2) have acute respiratory infections (e.g., acute bronchitis, acute upper respiratory tract infection, pneumonia) causing sputum production and coughing, and (3) can be monitored daily. Key exclusion criteria include, (1) recent treatment with expectorant medications within 24 h of randomization, (2) chronic lung conditions such as bronchial asthma, interstitial lung disease, or bronchiectasis, (3) significant chronic heart, brain, gastrointestinal, renal, hematologic, endocrine, or other systemic disease, (4) AST or ALT >1.5 times upper limit of normal or total bilirubin or serum creatinine > the upper limit of normal, and (5) poor adherence to drug regimen.

### Outcomes

The primary endpoint was the changes in cough symptom scores. Cough symptom scoring system was adopted from previously validated scoring systems (15, 16). The scores range from 0 to 5 with 0 being no cough and 5 being distressing cough that affects most daily activities. The full cough evaluation score is included in Supplementary Material 1. Day time coughing and nocturnal coughing were scored at baseline, during treatment day 1 and 4, and at the end of treatment. Patients’ guardians were provided with instructions on the scoring system and guardians scored the patients based on the provided descriptions.

Visual analog scale (VAS) was used to assess severity of cough. Patients’ guardians were asked to mark on a 0–100 mm VAS strip regarding their subjective evaluation of their children's cough severity with a score of 10 representing the most severe cough and a score of 0 representing absence of cough.

Quality of life assessment using the Parent-proxy Children's Acute Cough-specific Quality of Life (PAC-QoL) questionnaire was also done at baseline and the end of treatment. This 12-question questionnaire aims to assess the quality of life for parents of children with chronic cough. The score is used and validated in previous studies. However, one question was modified to better suit our patient population. Supplementary Material 2 includes the questions assessed by the PAC-QoL (17, 18).

Adherence to medication is assessed using the medication diary provided to the parents. Adherence assessment is based on the time of administration of medication and the amount of medication taken. Acceptable adherence is deemed to be administration within 2 min with little to no residual medication. Supplementary Material 3 include the full criteria adopted from previous literature. Poor adherence is deemed to be administration requiring more than 2 min and patients spitting out or refusing the medication or choking on the medication. Medication adherence observations are categorized into swallowing medication whole, taking medication with small amounts of residue, spitting out medication, choking on medication, and refusing to take medication (19, 20).

Safety outcome is recorded as adverse events (AEs) that occurred from day 1 of treatment to the end of treatment. All AEs were evaluated for the likelihood of association with ambroxol treatment.

### Statistical analysis

All data analyses were performed using SAS (Statistical Analysis Software 9.4, SAS Institute Inc, Cary, North Carolina, USA). Descriptive statistics such as mean, standard deviation, and frequency by treatment arm will be used to summarize the results. All analyses are done using recorded data. No missing values were imputed. All patients randomized and received at least 1 treatment dose was included in the analysis.

The study was designed to show non-inferiority for the primary endpoint in the modified intention-to-treat population. Based on past literature, the expected mean difference post treatment is 2 ± 0.5. With a one-sided significance level of 0·025, 73 patients in each group would be needed to reject a null hypothesis of inferiority with 85% power. After factoring in 20% patients loss to follow up, we estimated that we would need to follow approximately 88 patients in each arm (total 176 patients) to achieve power.

## Results

### Study patients

**Figure 1:**
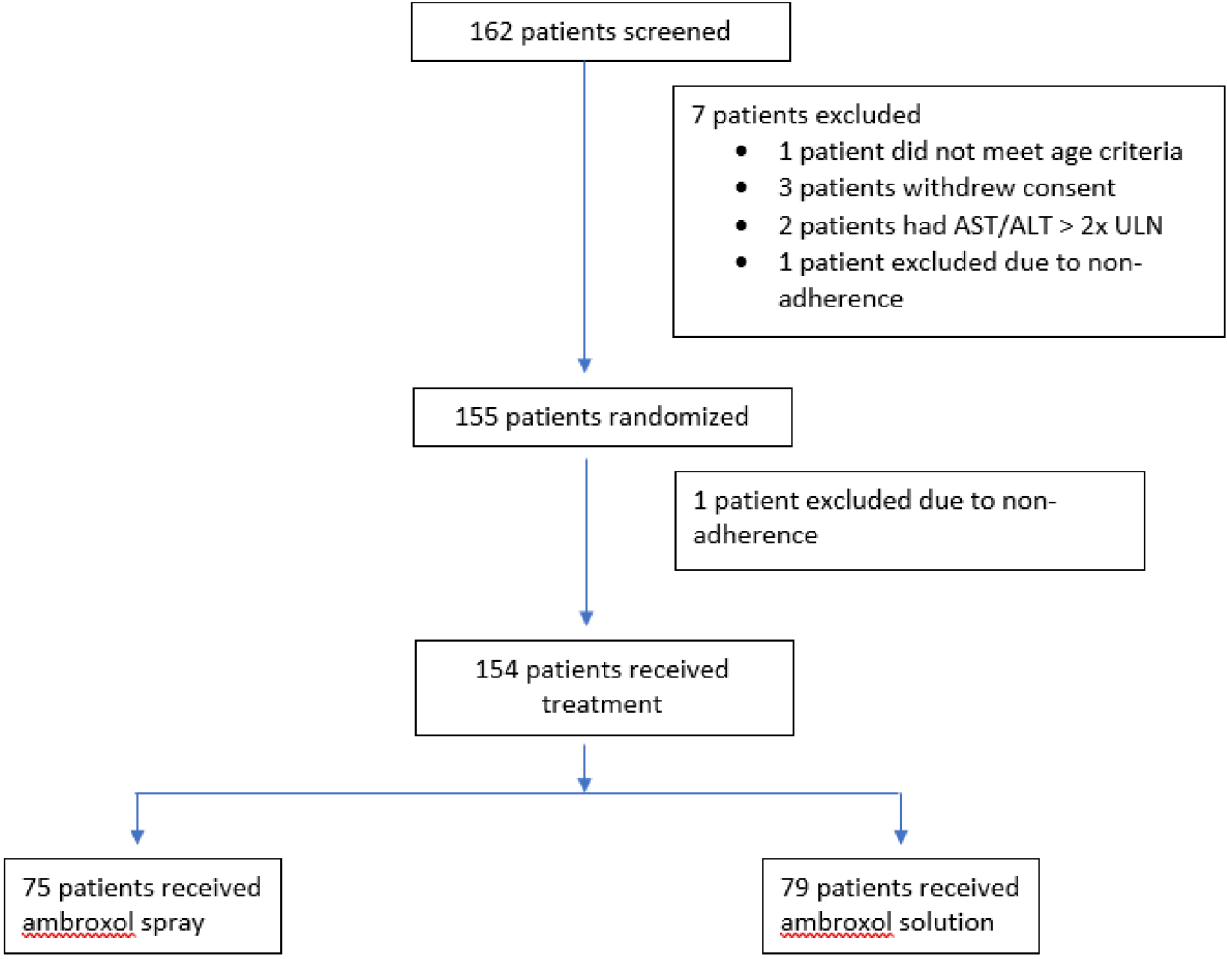
Process chart of application of inclusion criteria.

Between November 2021 and August 2022, a total of 162 patients were screened for study eligibility. Of these patients, 7 patients did not fulfill the criteria for randomization, and 1 patient was excluded after randomization due to non-adherence to the study regimen. Accordingly, 75 patients were randomly assigned to receive the ambroxol spray and 79 patients to receive the ambroxol solution for the modified intention-to-treat analysis (Figure 1). Demographic and baseline clinical characteristics were similar for the 2 groups (Table 1). Mean age of participants is 4 years old. Approximately 49% of participants had acute pneumonia with 57% of participants have one or more comorbidities. Most common concomitant medication class used is antibiotic medications with majority of patients receiving either azithromycin or cefotaxime.

**Table 1:** Characteristics of patients at baseline.

	Ambroxol spray (*N* = 75)	Ambroxol solution (*N* = 79)	*P*-value
Age, mean (SD), y	4.1 (1.4)	3.9 (1.4)	0.45
Female sex, *n* (%)	35 (46.67)	27 (34.18)	0.11
Height, mean (SD), cm	104.7 (11.5)	102.1 (10.0)	0.15
Weight, mean (SD), kg	17.89 (5.25)	16.71 (4.05)	0.15
Type of acute respiratory infection			0.37
- Acute bronchitis	21 (28.00)	27 (34.18)	
- Acute upper respiratory tract infection	13 (17.33)	12 (15.19)	
- Acute pneumonia	37 (49.33)	31 (39.24)	
- Other	4 (5.33)	9 (11.39)	
Time to first dose of medication from diagnosis, mean (SD), day	3.4 (5.4)	2.4 (1.5)	0.82
Time to first dose of medication from symptom onset, mean (SD), day	8.5 (7.8)	6.8 (5.5)	0.12
Medications at screening			
- Antibiotic medications, *n* (%)	63 (84.00)	67 (84.81)	0.89
- Antiviral medications, *n* (%)	15 (20.00)	18 (22.78)	0.67
- Inhaled corticosteroids, *n* (%)	46 (61.33)	49 (62.03)	0.93
- Bronchodilators, *n* (%)	42 (56.00)	40 (50.63)	0.50

### Primary outcome

At the end of treatment, the cough symptom score compared to baseline was significantly improved in the ambroxol spray arm compared to the ambroxol solution arm (Table 2). During the daytime, the mean value of the change from the baseline in the spray group was −2.4 (0.86), and that in the solution group was −2.1 (0.94) (*P* = 0.0004). The average change in nighttime score compared to baseline in the spray group was −2.3 (0.90), compared with −2.1 (0.96) in the solution group (*P* = 0.02). The total average change from the baseline in the spray group was −4.7 (1.54), an 81.26% decrease, compared with −4.2 (1.62), a 73.66% decrease, in the solution group (*P* = 0.0008).

**Table 2:** Primary outcome of cough score changes at day 1, day 4, and end of treatment.

Primary outcome: cough score	Ambroxol spray (*N* = 75)	Ambroxol solution (*N* = 79)	*P*-value
Daytime cough score			
Mean at baseline, mean (SD)	3.0 (0.72)	3.0 (0.84)	0.60
Change from baseline at day 1, mean (SD)	−0.1 (0.32)	−0.1 (0.41)	0.72
- Least-squares mean difference vs. solution arm (95% CI) — liters	−0.023 (−0.152,0.105)		
Change from baseline at day 4, mean (SD)	−1.7 (0.91)	−1.4 (0.85)	<0.01
- Least-squares mean difference vs. solution arm (95% CI) — liters	−0.157 (−0.268,−0.047)		
Change from baseline at end of treatment, mean (SD)	−2.4 (0.86)	−2.1 (0.94)	<0.01
- Least-squares mean difference vs. solution arm (95% CI) — liters	−0.336 (−0.521,−0.151)		
Nighttime cough score			
Mean at baseline, mean (SD)	2.7 (0.94)	2.7 (1.03)	0.61
Change from baseline at day 1, mean (SD)	−0.1 (0.41)	−0.2 (0.58)	0.93
- Least-squares mean difference vs. solution arm (95% CI) — liters	0.005 (−0.115,0.126)		
Change from baseline at day 4, mean (SD)	−1.6 (0.92)	−1.4 (0.78)	0.10
- Least-squares mean difference vs. solution arm (95% CI) — liters	−0.085 (−0.187,0.016)		
Change from baseline at end of treatment, mean (SD)	−2.3 (0.90)	−2.1 (0.96)	0.02
- Least-squares mean difference vs. solution arm (95% CI) — liters	−0.206 (−0.385,−0.028)		
Overall cough score			
Mean at baseline, mean (SD)	5.7 (1.52)	5.7 (1.74)	0.86
Change from baseline at day 1, mean (SD)	−0.1 (0.67)	−0.3 (0.94)	
- Least-squares mean difference vs. solution arm (95% CI) — liters	−0.019 (−0.232,0.194)		
Change from baseline at day 4, mean (SD)	−3.3 (1.53)	−2.8 (1.41)	<0.01
- Least-squares mean difference vs. solution arm (95% CI) — liters	−0.244 (−0.423,−0.065)		
Change from baseline at end of treatment, mean (SD)	−4.7 (1.54)	−4.2 (1.62)	<0.01
- Least-squares mean difference vs. solution arm (95% CI) — liters	−0.543 (−0.859,−0.228)		

### Secondary outcomes

At the end of the study, all secondary outcomes are statistically different comparing the spray and the solution arms (Table 3). At the end of treatment, the change from baseline regarding cough severity measured by VAS was −5.7 in the spray group and −5.2 in the solution group (*P* = 0.0064). Compared to baseline, the VAS decreased by 82.62% in the spray group compared to a 76.25% decrease in the solution group (*P* = 0.0064). The change from baseline regarding quality-of-life assessment measured using the PAC-QoL questionnaire was −16.4 (decreased by 69.09%) in the spray group and −12.4 (decreased by 51.08%) in the solution group (*P* < 0.0001). All parents in the spray group reported good adherence regarding easy administration of medication (within 2 min with little to no residual medication) compared to only 49% in the solution group (*P* < 0.0001). Good adherence, defined as swallowing all of the medications or taking medications with small amounts of residue, was reported in 100% of patients in the spray group compared to 81% in the solution group (*P* < 0.0001). A total of 97% of patients in the spray group was able to take all the medication on first attempt compared to 38% in the solution group (*P* < 0.0001).

**Table 3:** Secondary outcomes including cough severity at day 1, 4, and end of treatment, quality of life assessment, and medication adherence.

	Ambroxol spray (*N* = 75)	Ambroxol solution (*N* = 79)	*P*-value
Cough severity measured using VAS			
Mean at baseline, mean (SD)	6.8 (1.93)	6.8 (1.79)	
Change from baseline at day 1, mean (SD)	0.0 (0.56)	−0.2 (0.70)	0.82
- Least-squares mean difference vs. solution arm (95% CI) — liters	−0.033 (−0.323,0.257)		
Change from baseline at day 4, mean (SD)	−4.3 (2.32)	−3.9 (1.87)	0.03
- Least-squares mean difference vs. solution arm (95% CI) — liters	−0.276 (−0.520,−0.032)		
Change from baseline at end of treatment, mean (SD)	−5.7 (2.09)	−5.2 (2.04)	0.01
- Least-squares mean difference vs. solution arm (95% CI) — liters	−0.600 (−1.030,−0.170)		
Quality of life assessment measured using the PAC-QoL questionnaire			
Mean at baseline, mean (SD)	23.7 (7.67)	24.2 (9.11)	
Change from baseline at end of treatment, mean (SD)	−16.4 (9.14)	−12.4 (10.17)	<0.01
Acceptable adherence			
Good adherence, *n* (%)	75 (100.0)	39 (49.37)	<0.01
Medication adherence			
Good adherence, *n* (%)	75 (100.0)	64（81.01）	<0.01

### Safety outcomes

A total of 1 (1.33%) adverse event was reported in the spray group and 9 (6.33%) was reported in the solution group (Table 4). Out of the 9 adverse events reported in the solution group, 2 (2.53%) of the treatment-emergent adverse events were determined to be grade 3. Some patients had multiple adverse events reported. No serious adverse event was reported in the spray group.

**Table 4:** Safety outcomes characterizing adverse events comparing the two groups.

	Ambroxol Spray (*N* = 75) *n* (%)	Ambroxol Solution (*N* = 79) *n* (%)	*p*-value
Any TEAE	1 (1.33)	5 (6.33)	0.2105
Grade 3 TEAE	0	2 (2.53)	0.4971

TEAE, treatment-emergent adverse events.

## Discussion

Children are more prone to respiratory infections due to rich respiratory vessels, tender mucosa, and poor ciliary clearing ability. Children's bronchial cartilage is soft and lacks elastic tissue support, which leads to sputum built up. When the respiratory tract is infected by pathogens such as bacteria, viruses, and mycoplasma, the airway mucus hypersecretion is activated, and the mucus becomes thick. As a result, the airway is easily blocked. Excessive secretion of mucus can cause mucociliary system clearance dysfunction and local defense function damage, leading to airway obstruction and hard-to-treat infections. Therefore, it is crucial in treating respiratory tract infections to remove respiratory tract obstruction and expel phlegm to keep the respiratory tract unobstructed.

Expectorant drugs can be categorized into expectorants, mucolytics, mucokinetics, and mucoregulators based on their mechanisms of action (21). As a mucous motility agent, ambroxol is widely used in clinical practice. The pharmacological properties of ambroxol include secretolytic activity (promoting mucus clearance and expectoration), anti-inflammatory, anti-oxidant activity, local anesthetic effect, and mucokinetic and mucociliary effects (11, 22). Since it was first licensed to the market in 1978, ambroxol has been developed in a variety of dosage forms, such as intramuscular and intravenous solutions, tablets, syrups, capsules, granules, oral sprays, suppositories, and atomized solutions. Compared to adults, children have higher requirements for the accuracy of drug dosage form and dosage due to the imperfect development of liver and kidney function and poor medication compliance. In China, only ambroxol oral solution is currently approved for use in the pediatric population. Currently, no oral spray form of ambroxol has been studied in children. The safety and effectiveness of ambroxol oral liquid in clinical application have been widely confirmed (12, 13).

Because of its local anesthetic properties, ambroxol oral sprays are used for the symptomatic treatment of acute pharyngeal pain caused by pharyngitis or viral airway infections (23, 24). The objective of this study was to evaluate the efficacy, safety, and compliance of ambroxol hydrochloride spray and ambroxol hydrochloride oral solution in the treatment of acute respiratory infectious diseases in children.

This study is the first one to directly compare ambroxol oral spray to oral solution. Patients were randomized and study done in multiple locations. However, this study has several limitations. Due to the limited sample size, it is necessary to expand the sample size in the future to conduct more in-depth studies on the efficacy and safety of ambroxol hydrochloride spray in children with acute respiratory infections. Another limitation is the lack of pharmacokinetic and pharmacodynamic studies. It is unknown whether the clinical benefits demonstrated by the spray formulation is due to bypassing first pass metabolism and achieving higher concentration or simply due to improved adherence. Further pharmacokinetic and pharmacodynamic studies are indicated to explore the mechanism of such findings.

## Conclusion

The results of this study showed that the improvement of cough symptom score, cough severity score, and quality of life score in the ambroxol hydrochloride spray group were higher than those in ambroxol hydrochloride oral solution group, and the difference was statistically significant. The ambroxol hydrochloride spray group had higher drug acceptability and better compliance. There was no significant difference in drug safety between the two groups. Due to various factors, children's choice of medication is very limited. The results provide clinicians with another drug option. More extensive placebo-controlled studies are warranted.

## Data Availability

The raw data supporting the conclusions of this article will be made available by the authors, without undue reservation.
