# Supplementary material for: Ambroxol hydrochloride spray (Luo Runchang®) in the treatment of acute respiratory infectious diseases: a prospective, multicenter, open label, randomized controlled study
Source: Front Pediatr. 2024 Sep 5;12:1380189. doi: 10.3389/fped.2024.1380189 (PMC11410591; doi:10.3389/fped.2024.1380189)
Supplement: Supplementary file 1 [file Datasheet1.pdf]

Supplementary Material 1. Cough symptom score.

Score	Daytime	Nighttime
0	No cough	No cough
1	Cough for one to two short period	Cough on waking only or cough before falling asleep
2	Cough for more than 2 short periods	Awoken once or woken early due to coughing
3	Frequent cough not interfering with usual activities	Frequent waking due to coughing
4	Frequent cough interfering with usual activities	Frequent cough most of the night
5	Distressing cough most of the day	Distressing cough preventing any sleep

Supplementary Material 2. Parent-proxy Children's Acute Cough-specific Quality of Life (PAC-QoL) questionnaire.

1. Did you feel sorry for your child because of his/her cough?
2. Were you worried concerned about your child's cough becoming worse?
3. Were you worried/concerned about your child not sleeping well because of the cough?
4. Were you worried/concerned about whether you should take your child to a doctor or emergency ward because of his/her cough?
5. Were you awakened during the night because of your child's cough?
6. Were you worried/concerned about your child’s cough lasting a long time?
7. Were you worried/concerned about the cause of your child's cough?
8. Were you worried/concerned about the effects of your child's cough on him/her, including his/her daily activities such as feeding and schooling?
9. Were you worried/concerned about your child not being able to breathe again after coughing?
10. Were you worried/concerned about your child feeling tired because of the cough?
11. Did you feel tired or exhausted because of your child's cough?
12. Did your family have to make accommodations due to your child's cough (i.e., work leave, time off, minimize outing time/family activity time)?

Supplementary Material 3. Medication acceptance criteria

Criterion	Description
1	Everything swallowed: no liquid residuals found during oral inspection
2	Small runlet: liquid rinse or flowing out off the mouth
3	Spat out: no observed deglutition and the child disgorged the syrup directly
4	Choked on: some of the liquid was inhaled or a cough was caused during swallowing
5	Refused to take: all actions preventing the parent placing the dosing instrument into the mouth or intentionally closing the lips
